# A novel methanol-free *Pichia pastoris* system for recombinant protein expression

**DOI:** 10.1186/s12934-016-0578-4

**Published:** 2016-10-21

**Authors:** Wei Shen, Ying Xue, Yiqi Liu, Chuixing Kong, Xiaolong Wang, Mengmeng Huang, Menghao Cai, Xiangshan Zhou, Yuanxing Zhang, Mian Zhou

**Affiliations:** 1 State Key Laboratory of Bioreactor Engineering, East China University of Science and Technology, 130 Meilong Road, Shanghai, 200237 China; 2 Shanghai Collaborative Innovation Center for Biomanufacturing (SCICB), Shanghai, 200237 China

**Keywords:** Recombination protein expression, *AOX1* promoter, Dihydroxyacetone, *GUT1*, *DAK*, *Pichia pastoris*

## Abstract

**Background:**

As one of the most popular expression systems, recombinant protein expression in *Pichia pastoris* relies on the *AOX1* promoter (P_*AOX1*_) which is strongly induced by methanol. However, the toxic and inflammatory nature of methanol restricts its application, especially in edible and medical products. Therefore, constructing a novel methanol-free system becomes necessary. The kinases involved in P_*AOX1*_ activation or repression by different carbon sources may be promising targets.

**Results:**

We identified two kinase mutants: Δ*gut1* and Δ*dak*, both of which showed strong alcohol oxidase activity under non-methanol carbon sources. Based on these two kinases, we constructed two methanol-free expression systems: Δ*gut1*-Hp*GCY1*-glycerol (P_*AOX1*_ induced by glycerol) and Δ*dak*-DHA (P_*AOX1*_ induced by DHA). By comparing their GFP expression efficiencies, the latter one showed better potential. To further test the Δ*dak*-DHA system, three more recombinant proteins were expressed as examples. We found that the expression ability of our novel methanol-free Δ*dak*-DHA system was generally better than the constitutive *GAP* promoter, and reached 50–60 % of the traditional methanol induced system.

**Conclusions:**

We successfully constructed a novel methanol-free expression system Δ*dak*-DHA. This modified expression platform preserved the favorable regulatable nature of P_*AOX1*_, providing a potential alternative to the traditional system.

**Electronic supplementary material:**

The online version of this article (doi:10.1186/s12934-016-0578-4) contains supplementary material, which is available to authorized users.

## Background

Methylotrophic yeast refers to a limited number of yeast species which are able to utilize methanol as the sole carbon and energy source for cell growth. *Pichia pastoris*, *Hansenula polymorpha*, *Candida boidinii* and *Pichia methanolica* are the most typical examples [1, 2]. In order to metabolize methanol, these yeast species express an alcohol oxidase, named Aox in *P. pastoris*, Mox in *H. polymorpha* and Aod in *C. boidinii* [3]. *Pichia pastoris* has two alcohol oxidase coding genes, *AOX1* and *AOX2*. The strength of *AOX1* promoter (P_*AOX1*_) is much stronger than P_*AOX2*_ under methanol induction, therefore *AOX1* is the major source of methanol-oxidizing activity [4]. Besides, in methanol cultured *P. pastoris* cells Aox protein level could reach 30 % of total soluble proteins [5]. *Pichia pastoris* has been exploited as an excellent heterologous protein expression system in 1980s [1]. So far, over 5000 recombinant proteins have been successfully expressed in *P. pastoris* including insulin, α-interferon and hepatitis B antigen [6] (http://www.pichia.com/). The increasing popularity of this particular expression system could be attributed to the following reasons [1, 7, 8]: (1) The *P. pastoris* genome has been completely sequenced and a lot of genetic manipulation tools are available; (2) The culture condition is simple, and cells can do high-density culture with high levels of protein expressed at the intra- or extra-cellular level; (3) As a eukaryote, *P. pastoris* is able to perform special modifications such as glycosylation.

In most cases, recombinant protein expression is driven by P_*AOX1*_ in *P. pastoris*. P_*AOX1*_ is induced only by methanol and repressed by other carbon sources such as glucose, glycerol and ethanol [4]. This special induction and repression feature functions as a switch which turns recombinant protein expression on and off under different culture conditions. This is beneficial especially when expressing proteins that are toxic towards cell growth. However, this system also has limitations. Since P_*AOX1*_ induction requires methanol, this toxic and inflammable material needs special handling and is not suitable for producing edible and medical products [8, 9]. In addition, the by-product hydrogen peroxide (H_2_O_2_) of methanol metabolism brings oxidative stress, which may result in the degradation of recombinant proteins [10, 11].

One way to solve the problem is to develop a methanol-free expression system, which does not rely on methanol to induce the *AOX1* promoter. Since P_*AOX1*_ is activated by methanol and repressed by glucose and glycerol, interrupting the glucose/glycerol repression pathway, or activating the methanol activation pathway will be a good strategy. The activation or repression by carbon molecules towards P_*AOX1*_ is not direct, but rather through complicated signaling pathways which have not been fully understood yet. So far several protein factors have been reported to be involved in the alcohol oxidase gene promoter regulation. One example lies in the hexose transporter and sensor family. Gcr1 in *H. polymorpha* [9, 12] and Hxt1 in *P. pastoris* [8] are hexose transporters, and their mutation result in a de-repression of the alcohol oxidase promoter in glucose. As for hexose sensors, *H. polymorpha* Hxs1 [13] mutation and *P. pastoris* Gss1 mutation [14] cause de-repression of alcohol oxidase under glucose culture. Other existing studies focus on transcription factors. As shown in Table 1, a few transcription activators and repressors have been identified in methylotrophic yeasts. However, how the induction or repression signals are transduced from carbon molecules to these transcription factors is still largely unknown. Exploring these elements will be helpful to reveal more potential targets for constructing the methanol-free expression system.

**Table 1:** Summary of transcription factors of the alcohol oxidase promoter in three types of methylotrophic yeast

Organism	Factors	Classification	Reference
*H. polymorpha*	Mig1,2	Repressor	[31]
	Mut3	Activator	[32]
	Mpp1	Activator	[33]
*P. pastoris*	Nrg1	Repressor	[20]
	Mit1	Activator	[34]
	Prm1	Activator	[34]
	Mxr1	Activator	[35]
*C. boidinii*	Mig1	Repressor	[36]
	Trm1	Activator	[37]
	Trm2	Activator	[38]

Kinases always play an important role in cell signaling, since phosphorylation and de-phosphorylation processes are crucial for many biological activities. However, few kinases involved in P_*AOX1*_ activation/repression have been identified so far. Therefore we performed a kinase screening and identified two kinases named *GUT1* and *DAK*. By analyzing the phenotypes of the knocked out strains under different carbon sources, we constructed two strains whose *AOX1* promoter could be activated by glycerol or dihydroxyacetone (DHA) as sole carbon source. Then we discussed and tested the possibility for each of these strains to become a novel methanol-free system by expressing several recombinant proteins as examples.

## Results

### The Δ*gut1* and Δ*dak* strains have abnormal Aox activity or growth rates under different carbon sources

**Fig. 1:**
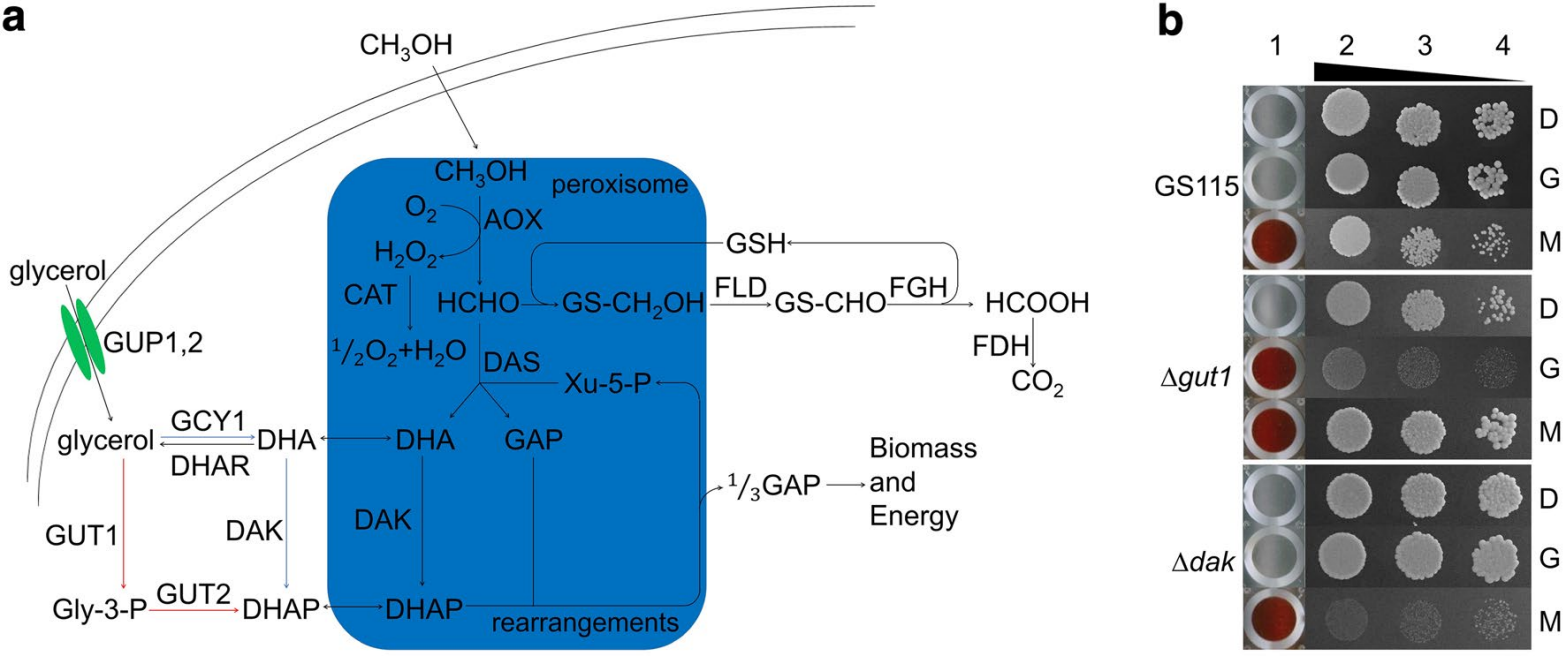
The Δ*gut1* and Δ*dak* strains have abnormal Aox activity or limited cell growth. **a** The outline of glycerol and methanol metabolism pathways in methylotrophic yeasts [3, 28, 30]. *Red arrows* indicate the phosphorylation pathway of glycerol usage while *blue arrows* indicate the oxidation pathway. **b** *Column 1* colorimetrical assay showing Aox activities in WT, Δ*gut1* and Δ*dak* strains under different carbon sources. *Deep red color* suggests high Aox activity. *White color* suggests no Aox activity. *Columns 2–4* spotting assay showing cell growth rates under different carbon sources. From *column* 2–4, spotting amount/concentration is 5 μL with 0.1, 0.01 and 0.001 OD_600_, respectively. *D* glucose; *G* glycerol; *M* methanol

In the genome of *P. pastoris*, 152 genes were annotated as kinase coding genes [15]. In order to screen for kinases involved in the P_*AOX1*_ activation/repression pathways, we knocked out 92 kinase genes separately and examined strain phenotypes under different carbon sources. For each knockout strain, a colorimetrical assay was used to measure the alcohol oxidase activity while cell growth was checked by the spotting assay. Among these 92 kinase genes, two members attracted our attention: *PAS_chr4_0783* and *PAS_chr3_0841*. *PAS_chr4_0783* encodes a glycerol kinase which converts glycerol to glycerol-3-phosphate, and *PAS_chr3_0841* is the gene of dihydroxyacetone kinase which converts DHA to dihydroxyacetone phosphate (DHAP) (Fig. 1a). Therefore *PAS_chr4_0783* is named Pp*GUT1* or *GUT1* here, and *PAS_chr3_0841* is named Pp*DAK* or *DAK*. As shown by Fig. 1b, Aox in the wild-type strain GS115 was induced by methanol and strictly repressed by glucose and glycerol. However, Aox expression was de-repressed in glycerol cultured Δ*gut1* strain although cell growth was also largely restricted. The Δ*dak* strain did not have any abnormality in Aox activity, but its growth under methanol was severely impaired (Fig. 1b).

### The Δ*gut1*-Hp*GCY1* strain has the potential to be developed to a novel methanol-free expression system

**Fig. 2:**
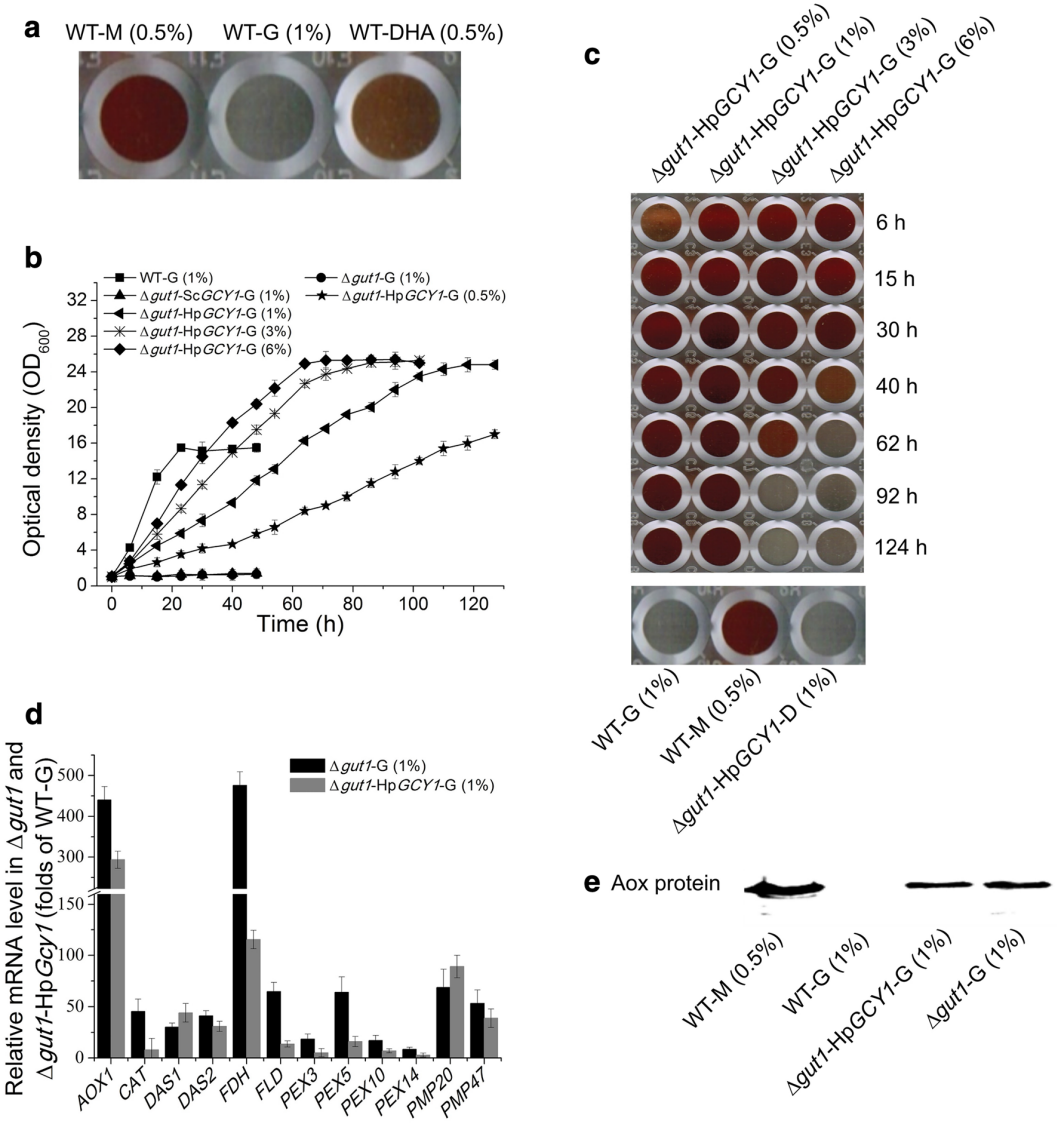
The Δ*gut1*-Hp*GCY1* strain has the potential to be developed to a novel methanol-free expression system. Concentrations of carbon sources were shown in brackets by the mass/volume percentage. *M* methanol; *G* glycerol; *D* glucose; *DHA* dihydroxyacetone. **a** Colorimetrical assay showing Aox activity in methanol, glycerol and DHA cultured wild-type strains. **b** Growth curves of WT, Δ*gut1*, Δ*gut1*-Sc*GCY1* and Δ*gut1*-Hp*GCY1* strains on different concentrations of glycerol. **c** Colorimetrical assay showing Aox activity of the Δ*gut1*-Hp*GCY1* strain under different glycerol concentrations and growth hours. Aox activity was not detected in glucose cultured Δ*gut1*-Hp*GCY1* strain. Higher Aox activity corresponds to *deeper red color* in the colorimetrical assay. **d** Q-PCR comparing the gene transcription levels in Δ*gut1* and Δ*gut1*-Hp*GCY1* strains grown on YNG medium. Folds were calculated towards the glycerol cultured WT strain. **e** Western blot showing the Aox protein levels of Δ*gut1* and Δ*gut1*-Hp*GCY1* strains grown on glycerol. Glycerol or methanol cultured WT strains served as negative and positive controls, respectively

Usually glycerol could be metabolized through two pathways in yeast, including the glycerol kinase mediated phosphorylation pathway and the glycerol dehydrogenase mediated oxidation pathway (Fig. 1a). As the first step of phosphorylation or oxidation pathway, glycerol is converted to glycerol 3-phosphate or DHA, respectively. Then both of them are converted to DHAP by different enzymes. Different methylotrophic yeasts prefer different pathways. For example, *C. boidinii* NO. 2201 utilizes the phosphorylation pathway and *Hansenula ofunaensis* prefers the oxidation pathway, while *H. polymorpha* has both [16]. As for *P. pastoris*, since Δ*gut1* showed significant Aox activity on glycerol but impaired cell growth (Fig. 1b), we considered that *P. pastoris* might preferentially use the phosphorylation pathway for the initial step of glycerol usage, and metabolites in this pathway may be repressing signals against Aox expression. These repressing signals are likely localized upstream of DHAP, since DHA and DHAP are common metabolites of both methanol and glycerol utilization pathways. Besides, DHA was an inducible carbon source and it supported Aox expression (Fig. 2a). Therefore, introducing the glycerol oxidation pathway into Δ*gut1* may be a good way to construct a methanol-free expression system. By converting glycerol directly to an inducible carbon source DHA, repressing signals generated by the phosphorylation pathway could be circumvented.

To test this idea, we introduced the glycerol dehydrogenases gene (*GCY1*) of *S. cerevisiae* and *H. polymorpha* into the Δ*gut1* strain separately, constructing the Δ*gut1*-Sc*GCY1 and* Δ*gut1*-Hp*GCY1* strains. As shown by Fig. 2b, Hp*GCY1* was able to rescue cell growth on glycerol while Sc*GCY1* could not. Besides, the growth rate of the Δ*gut1*-Hp*GCY1* strain increased with the elevated initial glycerol concentration, with a heavier final cell biomass at the stationary phase than WT. There results suggested that Hp*GCY1* works in *P. pastoris* to metabolize glycerol through the oxidation pathway while Sc*GCY1* does not. The reason why Sc*GCY1* did not work in *P. pastoris* may be due to the difference in conserved domains (Additional file 1: Figure S1). Usually, Sc*GCY1* works better in extreme conditions, such as high osmolality and micro-aerobic conditions [17, 18] while Hp*GCY1* works in both common and high osmolality conditions [19].

Then we checked the Aox activity in glycerol cultured Δ*gut1*-Hp*GCY1* strain (Fig. 2c). Aox activity could be detected in different glycerol concentrations from 0.5 to 6 %, suggesting P_*AOX1*_ was at least partially de-repressed in the Δ*gut1*-Hp*GCY1* strain. In addition, lower glycerol concentrations (0.5 and 1 %) supported longer Aox activity. Aox expression in the Δ*gut1*-Hp*GCY1* strain was still repressed by glucose (Fig. 2c, bottom). Therefore, this modified expression platform preserved the favorable regulatable nature of P_*AOX1*_.

The catabolism of methanol depends on both methanol utilization pathway (MUT pathway) and peroxisomes biogenesis [3]. Usually, the de-repression of Aox expression is accompanied by elevated activities of enzymes involved in MUT pathway and peroxisomes biogenesis [3, 20, 21]. These genes include *AOX1*, *CAT*, *DAS1*, *DAS2*, *FDH* and *FLD* in MUT, and *PEX3*, *PEX5*, *PEX10*, *PEX14*, *PMP20* and *PMP47* in peroxisome biogenesis. Therefore we checked the transcriptional levels of these genes in glycerol cultured WT, Δ*gut1*, and Δ*gut1*-Hp*GCY1* strains (Fig. 2d). Compared with the glycerol cultured WT strain, the transcriptional levels of these genes in Δ*gut1* and Δ*gut1*-Hp*GCY1* strains were much higher, especially *AOX1* and *FDH*. Significant amount of Aox protein could be detected in glycerol cultured Δ*gut1* and Δ*gut1*-Hp*GCY1* strains (Fig. 2e).

Taken together, these results indicate that P_*AOX1*_ de-repression under glycerol is at least partially achieved in the Δ*gut1*-Hp*GCY1* strain, and it has the potential to be developed to a novel methanol-free expression system. Here we named this system Δ*gut1*-Hp*GCY1*-glycerol.

### The Δ*dak* strain has the potential to be developed to a novel methanol-free expression system as well

**Fig. 3:**
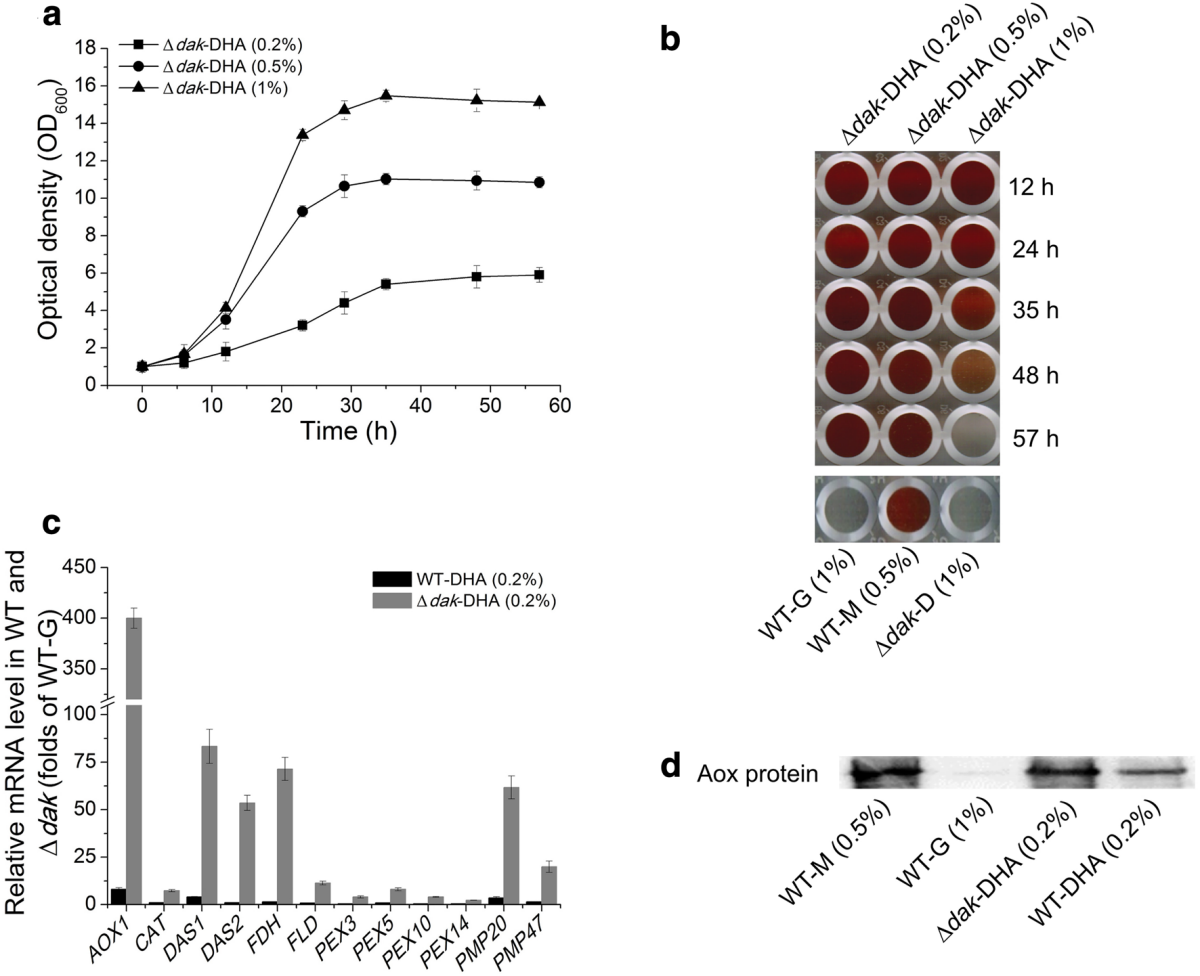
The Δ*dak* strain has the potential to be developed to a novel methanol-free expression system. Concentrations of carbon sources were shown in brackets by the mass/volume percentage. *M* methanol; *G* glycerol; *D* glucose; *DHA* dihydroxyacetone. **a** Growth curves of the Δ*dak* strain under different DHA concentrations. **b** Colorimetrical assay showing Aox activity of DHA cultured Δ*dak* strain. *Time points* indicate cell growth hours in DHA. Aox activity was not detected in glucose cultured Δ*dak* strain. Higher Aox activity corresponds to *deeper red color* in the colorimetrical assay. **c** Q-PCR comparing gene transcription levels in WT and Δ*dak* strains grown on DHA. Folds were calculated towards the glycerol cultured WT strain. **d** Western blot showing Aox protein levels in DHA cultured Δ*dak* strain. WT strains grown on methanol, glycerol and DHA serve as controls

Another interesting target revealed from our kinase screening is *DAK*. The Δ*dak* strain showed similar Aox activity profile as WT, but impaired cell growth under methanol (Fig. 1b). In 1998, Luers et al. deleted this gene in *P. pastoris* PPY4, and the strain growth was abolished by methanol but supported by DHA [22]. Since DHA is an inducible carbon source for P_*AOX1*_ in WT cell (Fig. 2a), we checked the effect of DHA on *P. pastoris* Δ*dak* strain. In agreement with previous studies, the Δ*dak* strain recovered growth on DHA and its growth rate increased with elevated DHA concentration (Fig. 3a).

The Aox activity in the Δ*dak* strain was then measured by the colorimetrical assay (Fig. 3b). The deep red color suggested that DHA was able to induce *AOX1* expression, and lower DHA concentrations (0.2 and 0.5 %) showed better Aox activities than higher DHA concentration, especially at the later growth stage. Aox induction here could also be switched on and off easily by changing carbon sources since it was strictly repressed by glucose (Fig. 3b, bottom). Therefore, this modified expression platform also preserved the inducible nature of P_*AOX1*_.

Again we examined the transcriptional levels of genes involved in MUT pathway and peroxisome biogenesis in DHA cultured WT and Δ*dak* strains. The transcriptional levels of these genes in DHA cultured Δ*dak* strain were much higher than that in DHA cultured WT strain (Fig. 3c). Being consistent with the colorimetrical assay, significant amount of Aox protein could be detected in DHA cultured Δ*dak* strain, which were comparable with that in methanol culture WT strain (Fig. 3d).

Taken together, these results suggested that the Δ*dak* strain also has the potential to be developed to a novel methanol-free expression system in which DHA functions as an inducible carbon source instead of methanol. This system is named Δ*dak*-DHA system.

### Compare the two potential novel methanol-free expression systems using GFP as a reporter

**Fig. 4:**
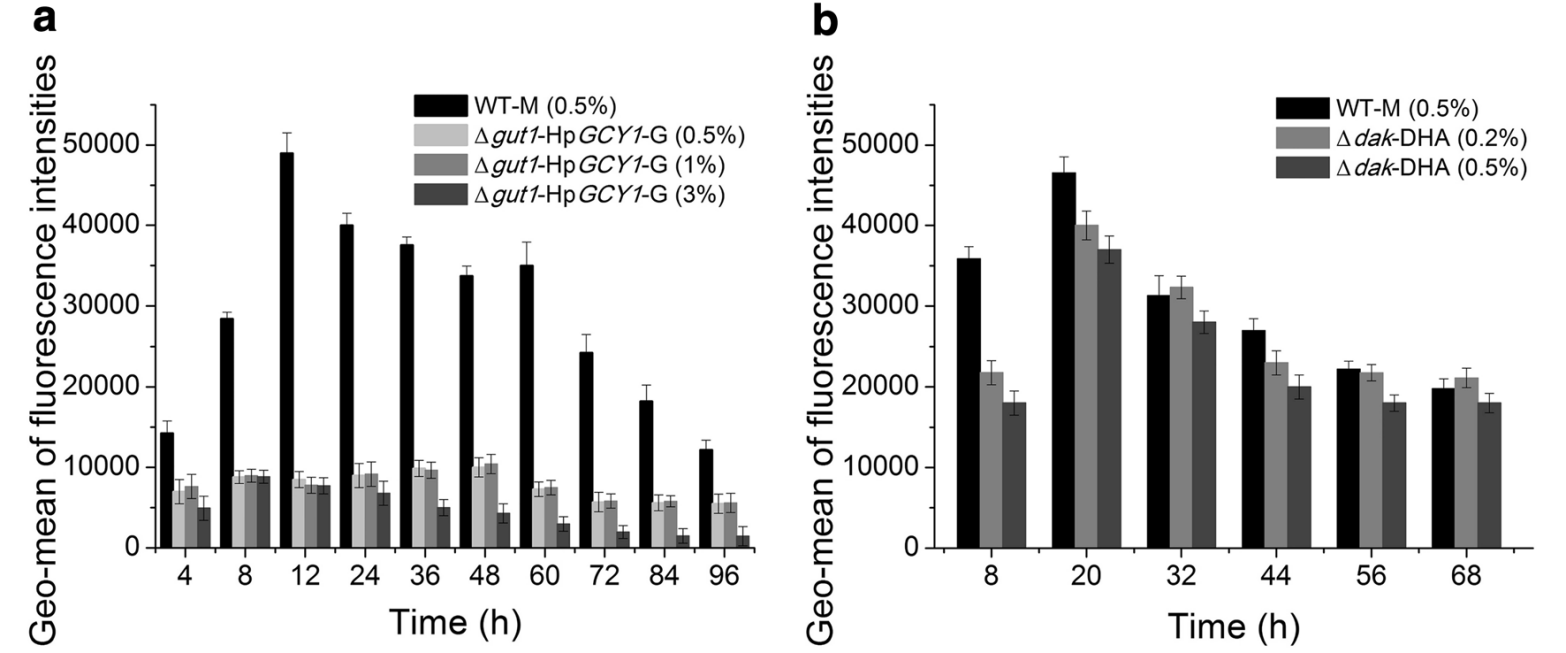
Evaluation of P_*AOX1*_ strength in Δ*gut1*-Hp*GCY1*-glycerol (**a**) and Δ*dak*-DHA (**b**) systems through GFP reporter assay. GFP intensity in methanol cultured WT strain is used as a reference here. Carbon source concentrations are shown in the *brackets* by the mass/volume percentage. *M* methanol; *G* glycerol; *DHA* dihydroxyacetone

In order to test the abilities of the two potential systems in recombinant protein production, we expressed GFP under P_*AOX1*_ in Δ*gut1*-Hp*GCY1*-glycerol system and Δ*dak*-DHA system. Green fluorescence intensity was measured to represent the GFP expression level, and GFP intensity in methanol cultured WT strain was used as a reference here. As shown by Fig. 4a, the fluorescence intensity of the Δ*gut1*-Hp*GCY1*-glycerol system was only 20–25 % of that from WT strain grown on methanol, while the Δ*dak*-DHA system showed 80–90 % (Fig. 4b). These distinct phenotypes could be traced to different growth rates between Δ*gut1*-Hp*GCY1* and Δ*dak* strains. Both glycerol and DHA are three-carbon molecules, however, the final cell density of glycerol cultured Δ*gut1*-Hp*GCY1* strain was much higher than that of DHA cultured Δ*dak* strain (Figs. 2b, 3a). Therefore, it is possible that Δ*gut1*-Hp*GCY1* converted more carbon sources into biomass, while Δ*dak* converted them into protein more efficiently. As a summary here, the Δ*dak*-DHA expression system seemed to function better than the Δ*gut1*-Hp*GCY1*-glycerol system, thus the former one was then selected for further study.

### Examine the recombinant protein production efficiencies in the Δ*dak*-DHA system

In order to further elucidate the potential of the Δ*dak*-DHA system, we expressed three more heterologous proteins and compared the expression levels with that in methanol induced WT strains. These heterologous proteins were amylase (Amy) from *Geobacillus* sp. 4j, glucose oxidase (God) from *Aspergillus niger* and hepatitis B small surface antigen (HBsAg) from human. Among them, Amy and God were secretory proteins while HBsAg was intracellular. Genes of these recombinant proteins were inserted after the *AOX1* promoter. In order to eliminate the influence of gene copy number, single copied expression cassette was selected for all strains. The widely used constitutive promoter P_*GAP*_ was also examined here as another control.

**Fig. 5:**
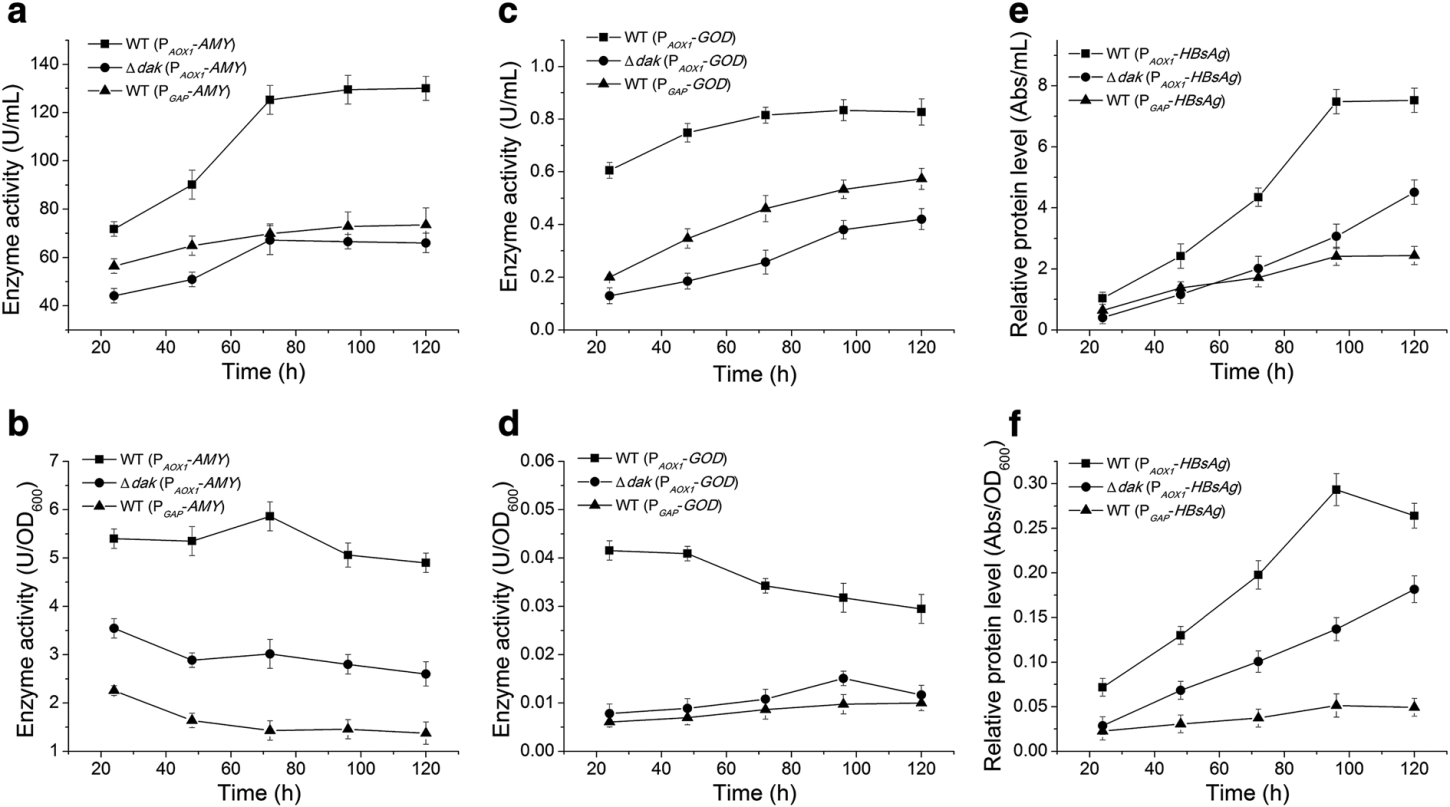
Expressional profiles of three heterologous proteins in the Δ*dak*-DHA system. The WT (P_*AOX1*_) system induced by methanol and the constitutive WT (P_*GAP*_) system serve as controls here. **a**, **b** The expressional profiles of amylase (AMY). **c**, **d** The expressional profiles of glucose oxidase (GOD). **e**, **f** The expressional profiles of hepatitis B small surface antigen (HBsAg)

As measured by enzyme activities, the expression levels of three recombinant proteins in the Δ*dak*-DHA system reached 50–60 % of methanol induced WT system, and became comparable (Amy and God) or even higher (HbsAg) than the constitutive P_*GAP*_ system (Fig. 5; Table 2). As shown by enzyme activity to biomass (U/OD_600_), the Δ*dak*-DHA system worked generally better than the constitutive P_*GAP*_ expression system, and showed about 50–60 % expression ability of the traditional methanol induced system.

**Table 2:** A summary of the protein expression levels in Fig. 5

Strain	Amy (U/mL)	God (U/mL)	HBsAg^a^ (Abs/mL)	Amy (U/OD_600_)	God (U/OD_600_)	HBsAg^a^ (Abs/OD_600_)
Δ*dak*-DHA (P_*AOX1*_)	67.18 ± 4.44	0.42 ± 0.03	4.51 ± 0.22	2.61 ± 0.12	0.012 ± 0.001	0.18 ± 0.01
WT-Methanol (P_*AOX1*_)	130.02 ± 5.32	0.83 ± 0.05	7.52 ± 0.43	4.90 ± 0.20	0.029 ± 0.002	0.26 ± 0.02
WT-Glucose (P_*GAP*_)	73.46 ± 4.32	0.57 ± 0.03	2.43 ± 0.19	1.37 ± 0.09	0.010 ± 0.001	0.049 ± 0.003

*Abs* absorbance

^a^Represented by relative enzyme activity

## Discussion

In this study we constructed and tested two modified *P. pastoris* expression systems on the basis of two kinase mutants. In both Δ*gut1*-Hp*GCY1*-glycerol and Δ*dak*-DHA systems, P_*AOX1*_ could be induced by non-methanol carbon sources (glycerol or DHA) and repressed by glucose. Between them, the Δ*dak*-DHA system showed better expression capacity. Exemplified by three typical recombinant proteins, its protein expression ability generally exceeded the constitutive P_*GAP*_ system, and reached 50–60 % of the traditional methanol induced system.

It should be noted that further optimization could be done to improve the protein expression efficiency in the Δ*dak*-DHA system. Since several transcription activators and repressors for P_*AOX1*_ are already identified, a combination strategy by overexpressing activators and knocking down repressors in the Δ*dak*-DHA system is worth trying. Besides, optimization of expression conditions and parameters in the Δ*dak*-DHA system will be likely to further increase the protein expression levels.

As the simplest ketose, DHA is always used as supplements in cosmetics, medicine and food industry [23]. DHA is non-toxic towards human and environment, and was added to FDA’s list of approved cosmetic ingredients in the 1970s. This novel methanol-free system will help to broaden the application of *P. pastoris* mediated recombinant protein expression, especially in producing medical and edible products.

Another interesting question attracted our attention is why the Δ*dak* strain grows on DHA but not methanol, since DHA is an intermediate in methanol metabolism (Fig. 1a). As one of the steps in methanol assimilation pathway, formaldehyde and xylulose 5-phosphate (Xu-5-P) are converted to one molecule of DHA and one molecule of glyceraldehyde 3-phosphate (GAP) (Fig. 1a). If DHA cannot be phosphorylated to DHAP, DHA will accumulate and cannot contribute to the regeneration of the C_1_-acceptor molecule Xu-5-P, which will not be sufficient for a continued function of the Xu-5-P cycle. Since methanol and its first step product formaldehyde are toxic to cells, delayed dissimilation may cause growth arrest. We found that addition of xylose recovered cell growth in methanol cultured Δ*dak* strain (Additional file 1: Figure S2A) and promoted methanol utilization (Additional file 1: Figure S2B). It is likely that Xu-5-P generated in xylose metabolism served as acceptor molecule for formaldehyde fixation and then made the cell recover the growth in methanol.

In order to examine why the Δ*dak* strain growth was supported by DHA, we examined the activities of several related enzymes (Additional file 1: Table S2). Dak activity was totally abolished in the knockout, suggesting that *P. pastoris* does not have any additional isozymes. Both WT and *DAK* mutant showed significant DHA reductase (Dhar) activity (Additional file 1: Table S2, last column). These results indicate that in DHA cultured *DAK* mutants, DHA may be reduced to glycerol first and then be metabolized through the phosphorylation pathway. In order to test this, we constructed a double mutant Δ*gut1*Δ*dak* in which glycerol phosphorylation were blocked by *GUT1* mutation. As expected, this strain failed to grow on both glycerol and DHA (Additional file 1: Figure S2C).

## Conclusions

Our results indicate that the Δ*dak*-DHA system is a novel methanol-free *P. pastoris* system for recombinant protein expression. The *AOX1* promoter in this system is induced by non-methanol carbon source DHA and repressed by glucose. The protein expression ability of this novel system generally exceeds the constitutive P_*GAP*_ system, and reaches 50–60 % of the traditional methanol induced system. Therefore, this modified expression platform has solved limitations caused by methanol usage and preserved the regulatable nature of P_*AOX1*_, making a potential alternative to the traditional system. Future studies are still needed to further increase the protein expression efficiencies in this system.

## Methods

### Strains and culture conditions

*Pichia pastoris* GS115 (invitrogen) was used as the wild-type (WT) strain. Unless indicated, *P. pastoris* strains were grown at 30 °C in YPD medium [1 % (w/v) yeast extract, 2 % (w/v) peptone, 2 % (w/v) glucose] or minimal YNB medium [0.67 % (w/v) yeast nitrogen base without amino acids] supplemented with different carbon sources, e.g., 1 % (w/v) glucose (YND), 1 % (w/v) glycerol (YNG), or 0.5 % (v/v) methanol (YNM). For solid media, agar was added to 2 % (w/v). Cell density (OD_600_) was determined spectophotometrically at the wavelength 600 nm. *Escherichia coli* TOP 10 cells were used for plasmid propagation. Primers used in this study were listed in Additional file 1: Table S1.

### Quantitative real-time RT-PCR (qPCR) analysis

The WT, Δ*gut1*, Δ*gut1*-Hp*GCY1* and Δ*dak* cells were pre-grown in YPD to OD_600_ of 2–8 and washed three times with sterile water. The washed cell pellets were transferred to YNG and YNDHA media. After cultured at 30 °C for 2.5 h, cell pellets were harvested and subsequently used to mRNA isolation. Genomic DNA was removed and cDNA was synthesized using ReverTra Ace qPCR RT Kit (TOYOBO). qPCR was carried out as described previously [8] using primers (Additional file 1: Table S1) designed by Beacon designer 7.9.

### Cell extract preparation and western blot analysis

To prepare cell extracts, 30–50 OD_600_ units of cells were harvested by centrifugation at 6000*g* for 3 min, washed twice with ice-cold 50 mM potassium phosphate buffer (pH 7.0), and then frozen at −20 °C. Cells were thawed and re-suspended in 1 ml lysis buffer [50 mM potassium phosphate buffer (pH 7.0), 1 mM phenylmethylsulfonyl fluoride (PMSF)]. Aliquots of 1 ml were mixed with 1.8 g glass beads (Biospec Products, Bartlesville, OK, USA) in a 2.0 ml screw-cap tube followed by disruption with a bead disrupter (Mini-BeadBeater-8; Biospec Products) for 8 cycles (1 min vibrating and 1 min resting in ice for each cycle). The lysate was centrifuged at 20,000*g* for 30 min, the pellet was discarded, and the supernatant was utilized for western blotting. The protein concentration was determined with a Bradford protein assay kit (Tiangen, Shanghai, China).

Each lane was loaded 10 μg total proteins for SDS-PAGE and then transferred onto a polyvinylidene difluoride (PVDF) membrane using the electrophoretic transfer method with rabbit anti-Aox antibody (a kind gift from Suresh Subramani, University of California, San Diego, USA) as the primary antibody and peroxidase-conjugated goat anti-rabbit immunoglobulin G as the secondary antibody.

### Construction of Δ*gut1*-Hp*GCY1* and Δ*gut1*-Sc*GCY1* strains

As Δ*gut1* has used *Sh ble* selection marker, we need new marker to construct the *GCY1* expression strains. We used primers ScaI-GAP/GAP-BamHI to amplify the *GAP* promoter from pGAPZA. pPIC3.5K was digested by restriction enzyme *Sca*I/*BamH*I to remove the *AOX1* promoter and then ligated with the *GAP* promoter which was digested by the same restriction enzymes. Finally, we got a new plasmid which contains the GAP promoter and the geneticin selection marker. Hp*GCY1* was amplified from *H. polymorpha* genome by using primers BamHI-HpGcy1/HpGcy1-NotI. Sc*GCY1* was amplified from *S. cerevisiae* genome using primers BamHI-ScGcy1/ScGcy1-NotI. After using restriction enzymes *BamH*I/*Not*I to digest the fragment HpGCY1, ScGCY1, and the above plasmid, the two fragments were ligated into the plasmid respectively. Then we obtained two plasmid P_*GAP*_-HpGCY1 and P_*GAP*_-ScGCY1. The two plasmids were linearized by *Sal*I and transformed into Δ*gut1* strain by electroporation. The positive transformants Δ*gut1*-Hp*GCY1* and Δ*gut1*-Sc*GCY1* were selected with histidine self-synthesis ability.

### Construction of WT-GFP, Δ*gut1*-Hp*GCY1*-GFP, and Δ*dak*-GFP strains

The primers 5-PBR-AOXTT/PBR-AOXTT-3 were used to amplify the fragment PBR-AOXTT [including three parts: *E. coli* origin of replication pBR322, Ampicillin resistance gene, and green fluorescent protein (GFP) expression cassette] from the plasmid pP-GFP. The primers 5-hph/hph-3 were used to amplify the hygromycin B phosphotransferase expression cassette from the plasmid pAG32, which was kindly provided by Prof. Suresh Subramani. These two fragments were ligated by using ClonExpress MultiS One Step Cloning Kit and then transformed into *E. coli* TOP 10 to screen correct plasmid. After verified by sequencing, correct plasmid was linearized by *Sac*I and transformed by electroporation into GS115, Δ*gut1*-Hp*GCY1*, and Δ*dak* respectively. The single copy strains of GFP expression cassette were screened according to the previously described method [24].

### Assays of yeast growth, Aox activities and GFP expression

The strains were pre-grown in YPD media to OD_600_ of 2–8. The cells were harvested by centrifugation at 3000*g* for 5 min, washed three times with sterile water, and resuspended with initial OD_600_ of 1.0 in 50 mL YNB media supplemented with various carbon sources. At suitable intervals, OD_600_ was measured for growth curve, 1 mL aliquot of culture media was removed, and cells were harvested by centrifugation and then stored at −80 °C for colorimetrical assay of Aox activities or measurement of GFP.

The reaction buffer of colorimetrical assay including 0.05 % (w/v) O-dianisidine, 0.15 % (w/v) CTAB, 1 % (v/v) methanol, 3 U/mL HRP, and 100 mmol/L potassium phosphate buffer (pH 7.5) [25]. When reacting, frozen cells were thawed and added 800 μL reaction buffer to incubate for about 20 min. Then 100 μL mixtures were transferred into 96-well plates and scanned into images by scanner.

For measuring GFP, frozen cells were thawed, washed twice with sterile water, and transferred into 96-well plates with diluting to about OD_600_ = 1. OD_600_ and GFP were measured by enzyme-labeled instrument (BioTek) with three biological replicates.

### Construction of three heterologous proteins expression strains

The GOD ORF was amplified from plasmid RINA1297-GOD (kindly provided by Juan Zhang, Jiangnan University) with primers SnaBI-GOD/GOD-NotI or KpnI-GOD/GOD-NotI. The fragment was digested by *SnaB*I/*Not*I and ligated into vector pPIC9K opened with the same restriction enzymes to yield the expression vector pPIC9K-GOD. With *Kpn*I/*Not*I, the GOD ORF was ligated into pGAPZαA to yield the expression vector pGAPZαA-GOD. The vector pPIC9K-GOD was linearized with *Pme*I and transformed by electroporation into WT and Δ*dak*. The vector pGAPZαA-GOD was linearized with *Bln*I and transformed into WT. In order to measure the recombinant proteins expression ability of the three expression systems, and eliminate the influence of copy number, single copy strain of God expression cassette was screened according to the previously described method [24]. The three single copy God expression strains were called WT (P_*AOX1*_-*GOD*), Δ*dak* (P_*AOX1*_-*GOD*) and WT (P_*GAP*_-*GOD*), respectively.

The construction process of Amy expression strains was just similar to strains WT (P_*AOX1*_-*GOD*), Δ*dak* (P_*AOX1*_-*GOD*) and WT (P_*GAP*_-*GOD*). The three single copy Amy expression strains were called WT (P_*AOX1*_-*AMY*), Δ*dak* (P_*AOX1*_-*AMY*) and WT (P_*GAP*_-*AMY*), respectively.

The HBsAg sequence was synthesized by Suzhou GENEWIZ biotech Co., Ltd., China. Primer pairs BamHI-HBsAg/HBsAg-NotI were used to amplify the sequence and the 680 bp product was digested with *BamH*I/*Not*I. It was then ligated into pPIC3.5K opened with the same restriction enzymes to yield the expression vector pPIC3.5K-HBsAg. With *BspT104*I/*Not*I, the HBsAg was ligated into pGAPZαA to yield the expression vector pGAPZ-HBsAg. By using *BspT104*I/*Not*I, the α-Factor secretion signal in pGAPZαA can be removed. The vector pPIC3.5K-HBsAg was linearized with *Sal*I and transformed by electroporation into WT and Δ*dak*. The vector pGAPZ-HBsAg was linearized with *BspH*I and Zeocin was used to select the positive transformant. The three single copy HBsAg expression strains were called WT (P_*AOX1*_-*HBsAg*), Δ*dak* (P_*AOX1*_-*HBsAg*) and WT (P_*GAP*_-*HBsAg*), respectively.

### Production and activity assays of three recombinant proteins

The strains WT (P_*AOX1*_-*GOD*), Δ*dak* (P_*AOX1*_-*GOD*) and WT (P_*GAP*_-*GOD*) were pre-grown in YPD media at 30 °C, 200 rpm. When OD_600_ reached 2–8, cells were harvested by centrifugation at 3000*g* for 5 min, washed three times with sterile water, and resuspended with initial OD_600_ 1.0 in 50 mL BMMY, BMDHAY and BMDY, respectively. The initial concentration of carbon source in three media was 0.5 % methanol, 0.2 % DHA, and 2.5 % glucose. Every 24 h after the shift, 1 mL aliquot of culture media was removed, and cells were separated by centrifugation (2 min at 8000*g*). PMSF was added to the culture supernatants to the final concentration of 1 mM to inactivate proteases, and samples were stored frozen at −20 °C for subsequent analysis. Cells biomass was also monitored during the course of cultivation. Methanol and DHA were fed every 24 h to keep the concentration around 0.5 and 0.2 %, respectively. The strains WT (P_*AOX1*_-*GOD*) and Δ*dak* (P_*AOX1*_-*GOD*) were induced for 120 h. The strain WT (P_*GAP*_-*GOD*) was batch culture for 120 h. God activity was measured using the coupled o-dianisidine-peroxidase reaction. It was determined as in Bankar’s manuscript [26].

The culture condition of Amy expression strains followed the same procedure of God. Amy activity was measured using the DNS method [27].

The culture condition of HBsAg expression strains followed the same procedure of God. The difference is that cells were harvested instead of the culture supernatants. HBsAg relative concentration was measured by HBsAg ELISA kit (Kehua, Shanghai, China).

### Enzyme activities assays for Dak, Gcy1 and Dhar

Assays were performed as described [28, 29]. Enzyme activities were examined by either NADH production or consumption. NADH level change was measured by UV absorbance at 340 nm.

## Additional file

**Additional file 1.** Additional files and tables.

## Abbreviations

Aox: alcohol oxidase
DHA: dihydroxyacetone
DHAP: dihydroxyacetone phosphate
MUT: methanol utilization pathway
Amy: amylase
God: glucose oxidase
HBsAg: hepatitis B small surface antigen
Dhar: DHA reductase
