# Supplementary material for: A novel methanol-free Pichia pastoris system for recombinant protein expression
Source: Microb Cell Fact. 2016 Oct 21;15:178. doi: 10.1186/s12934-016-0578-4 (PMC5073731; doi:10.1186/s12934-016-0578-4)
Supplement: Supplementary file 1 — Additional file 1. Additional files and tables. [file 12934_2016_578_MOESM1_ESM.pdf]

Supplementary Fig legends

Fig. S1. The distinct domains of Sc*GCY1* and Hp*GCY1*.

Fig. S2. The simple explain of Δ*dak* which could not grow on methanol but on DHA.

(A) The growth curves of WT and Δ*dak* strain on xylose or methanol alone and mixture of xylose and methanol. (B) Measurement of residual methanol percentage in cultures with and without xylose addition. (C) The growth curves of Δ*dak* and Δ*gut1*Δ*dak* double knockout strain cultured under indicated carbon sources.

Figure S1

ScGCY1

HpGCY1

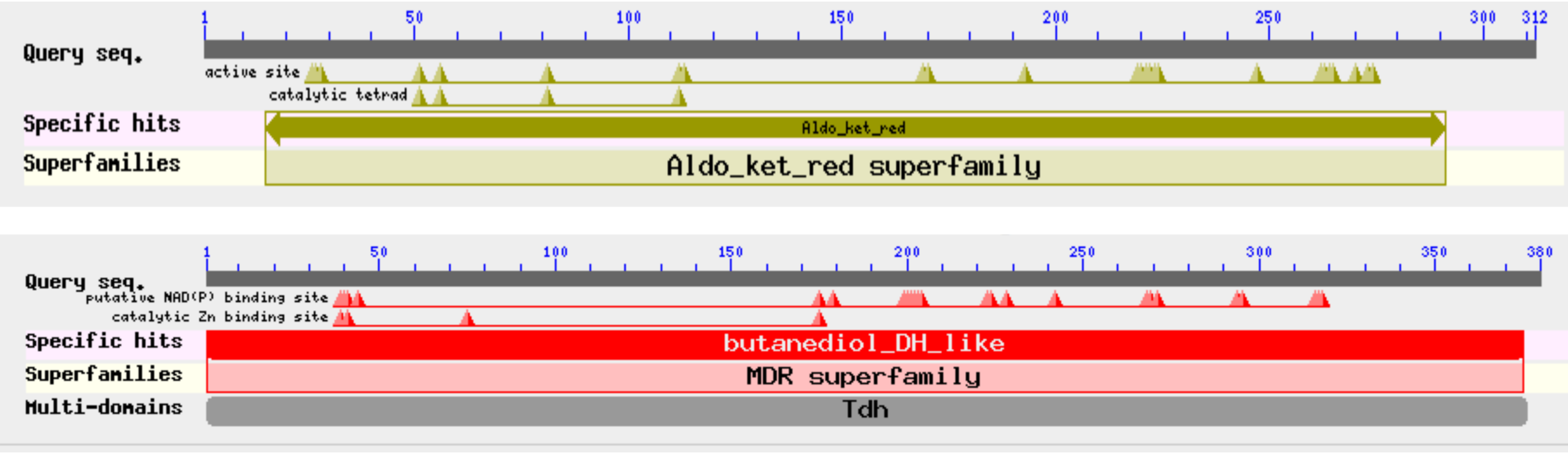

Figure S2

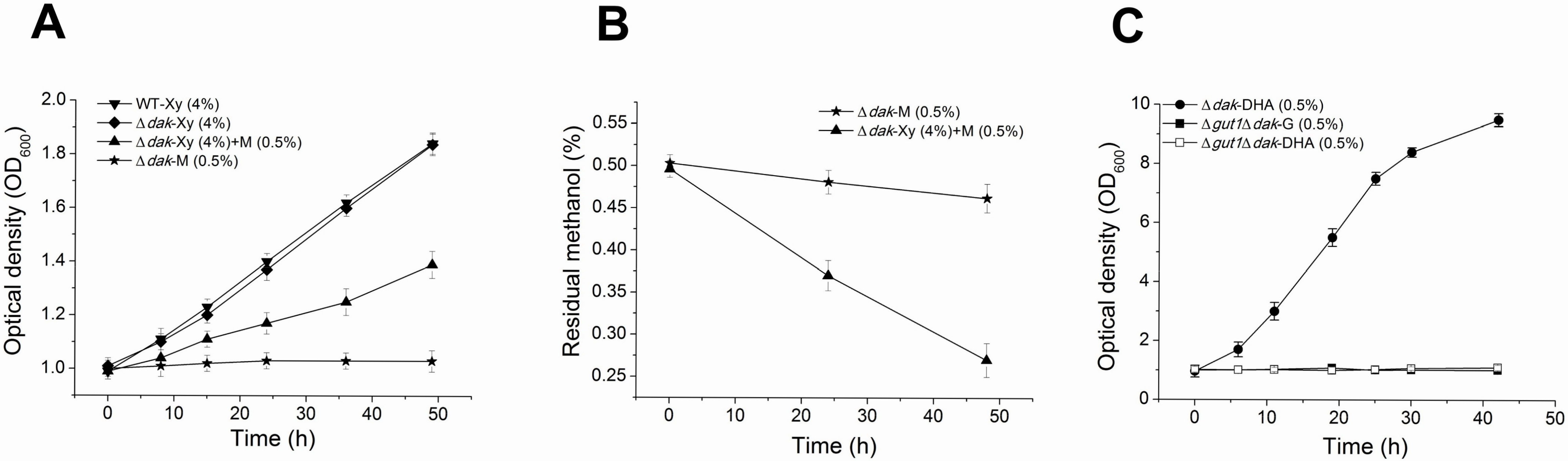
A
B
C
Optical density (OD_{600})
WT-Xy (4%)
Δ*dak*-Xy (4%)
Δ*dak*-Xy (4%)+M (0.5%)
Δ*dak*-M (0.5%)
Time (h)
Residual methanol (%)
Δ*dak*-M (0.5%)
Δ*dak*-Xy (4%)+M (0.5%)
Time (h)
Optical density (OD_{600})
Δ*dak*-DHA (0.5%)
Δ*gut1*Δ*dak*-G (0.5%)
Δ*gut1*Δ*dak*-DHA (0.5%)
Time (h)

Supplementary Table 1 Oligonucleotide primers used in this study.

Primer	Sequences (5’-3’)[a]
ScaI-GAP	AACGAGTACTAGATCTTTTTTGTAGAAAT
GAP-BamHI	CGCGGGATCCATAGTTGTTCAATTGATTGA
BamHI-HpGcy1	CGCGGATCCACCATGGGTAAAGGTTTACTTTATTACGGTAC
HpGcy1-NotI	ATAGTTTAGCGGCCGCTTAGGAAACCTCGTTCGGC
BamHI-ScGcy1	CGCGGATCCACCATGGGTCCTGCTACTTTACATGATTCTA
ScGcy1-NotI	ATAGTTTAGCGGCCGCTTACTTGAATACTTCGAAAGGA
5-PBR-AOXTT	AAACGAGCTCATGTGAGCAAAAGGCCAG
PBR-AOXTT-3	GCGGGGACAAGGCAAGCAAGCTTGCAC
5-hph	GTGCAAGCTTGCTTGCCTTGTCCCCGC
hph-3	TTGCTCACATGAGCTCGTTTTCGACACTG
SnaBI-GOD	TCCTACGTAAATGGCATTGAAGCCAGCCT
GOD-NotI	CTTGCGGCCGCTCACTGCATGGAAGCATAATCTTC
KpnI-GOD	TCGGGGTACCAATGGCATTGAAGCCAGCCT
XhoI-AMY	ATCGCTCGAGAAAAGAGCTGCTCCATTCAACGGAAC
AMY-NotI	CTTGCGGCCGCTTATGGCCAAGCAACCAATCT
BamHI-HBsAg	CGCGGATCCATGGAGAACATCGCATCAG
HBsAg-NotI	AATCGCGGCCGCTTAAATGTATACCCAAAGACAAA
BspT104I-HBsAg	ATCGTTCGAACCATGGAGAACATCGCATCAG
RT-ATP2F	TGATTAACAACATTGCTAAG
RT-ATP2R	AATAACACCAGTCTCCTT

RT-AOX1F	GAGGCCAGAGCCTTGGAA
RT-AOX1R	CCTTCGTTCTTTGCAGTT
RT-DAS1F	GGTGACGAGTTAGTAAAG
RT-DAS1R	CCTCTAACACGAGAAAGG
RT-DAS2F	GGTGATCAACTAGTTGCT
RT-DAS2R	CCTCTAATACGGGCCTTT
RT-CATF	GCTAATCACGCTAACAAT
RT-CATR	GGCAAGAGAATCAATCAA
RT-FLDF	TTGGTTCAGGACTATCTT
RT-FLDR	GTAATCACAGCACGAATA
RT-FDHF	TTCCACTCCATTCCATCC
RT-FDHR	CAACGACCAACAACTTCA
RT-PEX3F	ATCCTTGCTGATTGTATT
RT-PEX3R	CACCATTAGACTCTTGAT
RT-PEX5F	GGCTCGCTATAATCTTGG
RT-PEX5R	CAACACCTTCAACCTCAT
RT-PEX10F	GGCAGTTCTATAACATATCC
RT-PEX10R	CCTCCTAGTAATTCGTAGT
RT-PEX14F	GAGGTTAAGGAGGCATTG
RT-PEX14R	AGAATACGATGACACTTGG
RT-PMP20F	TTCCACATTCCATCATCT
RT-PMP20R	GCAACGACAATAAATCTCT

RT-PMP47F	AACAACCTACCAACACTAT
RT-PMP47R	GCTGATACCAACTCTTCT

[a] The underlined nucleotide sequences are for restriction enzyme recognition sites.

Supplementary Table 2 Enzyme activities in cell-free extracts of different strains cultured in DHA (0.5%, w/v).

strain	Enzyme activity ($nmol \cdot min^{-1} \cdot mg^{-1}$)		
	Dak	Gcy1	Dhar
WT-DHA	53.2±4.7	40.1±2.8	440.6±10.4
Δ*dak*-DHA	0	38.4±3.1	407.5±8.9
